# The influence of spousal care on the health of middle-aged and older adult caregivers in China—empirical analysis based on CHARLS data

**DOI:** 10.3389/fpubh.2025.1496637

**Published:** 2025-02-06

**Authors:** Rui Wang

**Affiliations:** School of Labor and Human Resources, Renmin University of China, Beijing, China

**Keywords:** spousal care, physical health, mental health, weekly exercise, non-food expenditure

## Abstract

**Objectives:**

With the decrease of the number of children and the increase of population mobility, child care presents a phenomenon of “double shortage” of human and time resources, and spousal care is becoming an important care source for middle-aged and older adult people in China. However, less studies have taken middle-aged and older adult couples as research subject to explore the health effects of spousal care in the process of aging. This paper attempts to explore the effects of caregiving on the health of caregivers from the perspective of spousal care.

**Methods:**

Using China Health and Retirement Longitudinal Study (CHARLS) database, this paper constructs a panel data fixed effect model to explore the effects of spousal care on the health of middle-aged and older adult caregivers. Further, instrumental variables (IV) estimation and propensity score matching (PSM) are used to minimize the impacts of endogeneity.

**Results:**

Spousal care has no significant effect on the physical health of middle-aged and older adult caregivers, but it has a significant negative effect on their mental health. Providing spousal care has a stronger negative effect on the mental health of female or rural middle-aged and older adult caregivers. The mechanism analysis shows that spousal care affects the mental health by reducing the number of weekly exercise and increasing the family’s non-food expenditure.

**Conclusion:**

The provision of spousal care in the middle-aged and older adult groups does harm the mental health of caregivers. Vulnerable groups such as families in which both spouses need care, female or rural caregivers expect more attention and support.

## 1 Introduction

With the development of economy and the improvement of medical level, population aging is becoming a major issue for every country around the world. According to the data released by National Bureau of Statistics, by the end of 2023, China’s older adult population aged 60 years old and above reached 296.97 million, accounting for 21.1 percent of the total population; of these, 216.76 million aged 65 years old and above, accounting for 15.4 percent of the total population. The rise in both the absolute quantities and relative proportions of the older adult population has placed higher and more urgent demands on older adult care. While the total numbers and proportion of the older adult population are rising, “long-term survival with illness” has become a health challenge in China’s “longevity era” (1).

Population aging is a strategic issue related to the long-term development of economy and society. In October 2020, the Fifth Plenary Session of the 19th Central Committee of the Communist Party of China (CPC) proposed the “implementation of a national strategy for actively coping with population aging,” which elevated the issue of actively coping with population aging to the level of a national strategy for the first time. Data released by National Health Commission of the People’s Republic of China in 2018 showed that the number of disabled and semi-disabled older adult people in China increased to 44 million, while the total number of older adult people with chronic diseases reached 150 million. As the degree of aging deepens and the proportion of disabled, semi-disabled and sick older adult people increases, the demand for older adult care services also grows. And issues related to older adult care are increasingly becoming a hot topic in both society and academia (2). Achieving a high and balanced level of older adult care is an important element in the implementation of the national strategy for actively responding to the population aging (3).

Care work includes unpaid care work and paid care work, which are interchangeable to some extent. The cost of the former is the cost of the family member’s time, while the cost of the latter is the price of formal care (4). The vast majority of care needs are provided by informal unpaid carers throughout the world (5, 6). It is estimated that unpaid care work is equivalent to the value created by two billion carers worldwide working unpaid for 8 h a day, which is equivalent to 5% of total global GDP (5). The Chinese government currently promotes a “90–7–3” model in older adult care, which means 90% of older adult people receive care at home, 7% receive care from the community, and only 3% receive care in nursing homes or similar institutions (7); regarding informal family care as the most important way to meet the care needs of older adult people.

Traditionally, there is a care model of “Raise children to provide against old age” in China. However, on the one hand, One-Child Policy since the 1980s in China has brought about a decrease in the number of children and a reduction in average family size (8). On the other hand, with the development of industrialization and urbanization, more and more young people go to big cities to work, and the phenomenon of intergenerational cohabitation has been reduced. In rural and urban–rural areas, more and more older adult people have been left behind (7). There is a “double shortage” of human and time resources for child care. Similar to many Organization for Economic Co-operation and Development (OECD) countries, spouses are becoming the main source of older adult care in response to the “care crisis” now and in the future (9, 10).

When it comes to the care for middle-aged and older adult people in China, the existing research focuses more on intergenerational perspective, exploring the impact of caregiving on the health of adult children (11–13) or on the health of older adult people who are looked after (14, 15). These studies ignore the role that spouses may play and how they may be affected. What is the effect of caregiving on spouses who are also middle-aged and older adult people? Does it have a negative effect on their health? Using China Health and Retirement Longitudinal Study (CHARLS) database, this paper attempts to explore the effects of caregiving on the health of middle-aged and older adult caregivers from the perspective of spousal care and further explores the possible mechanism pathways of this effect. The research of this paper about the health effects of spousal care will add to supplements on the effects of informal care based on Chinese situation theoretically. In practice, it will increase awareness of the “care crisis” and provide a factual basis for the implementation of policies to promote the sustainable development of informal care.

This paper consists of five sections. In the following section, we review the literature and put forward the research hypotheses. Section three describes the data source and the methods of this paper. Section four reports the results. This paper ends with a discussion of the results and some policy implications drawn from them.

## 2 Literature review and research hypothesis

### 2.1 Literature review

Care Economics is a branch of economics that studies the production, distribution and consumption of care services in family, market and government with the analytical paradigm of economics and how to realize the optimal allocation of care services in the above fields to meet the needs of social groups (16). Care work is divided into formal care work and informal care work. Informal care is currently the main form of care for the older adult people in China (17).

In addition to the effect of informal care on the health and wellbeing of care recipients, the effect of informal care on the health of caregivers is also an issue worthy of concern and exploration. Existing research suggests that there may be a “spillover effect” of informal care on caregivers (18). This “spillover effect” can be further differentiated into the “caregiving/caregiver effect” and the “family effect.”

The “caregiving/caregiver effect” refers to the welfare effect of providing informal care. For example, in caring for a patient, there is an indirect and significant effect of the patient’s illness and dependence on care on the welfare of the informal caregivers (19). The “family effect” (20) refers to the effect of caregivers caring for the health of their family members (e.g., their children and parents) (18, 21). This effect means that the level of health of the patient has a direct effect on the level of wellbeing of family caregivers (22).

Existing research on the effect of caregiving on the health of caregivers focuses more on intergenerational relationships, exploring the effect of children caring for their parents on their own health; or from the perspective of “grandparents caring for grandchildren,” which is unique in China, exploring the effect of intergenerational caregiving on the health of middle-aged and older adult people. Longitudinal data combined with instrumental variable analyses for the United States, Korea, and 13 countries in Europe, respectively, showed that caring for parents or parents-in-law has varying degrees of negative effects on the health of adult children. The extent of the negative effect is highly heterogeneous by gender and marital status (11–13). The findings of existing research about “grandparents caring for grandchildren” on the health of middle-aged and older adult people vary somewhat. Moderate “grandparents caring for grandchildren” among young older adult people is beneficial to their physical health and psychological wellbeing (23, 24). However, studies have also found that it has a significant negative effect on the physical and psychological health for rural grandparents (25).

In summary, existing research on caregiving about middle-aged and older adult people have more often explored the effects of children caring for their parents or grandparents caring for grandchildren on the health of caregivers from the perspective of intergenerational relationships; and less often have taken middle-aged and older adult couples as research subject to explore the health effects of spousal care in the process of aging. Therefore, Firstly, based on existing research, this paper explores the effect of care on caregivers’ health from the perspective of spousal care in the middle-aged and older adult groups, providing a supplement for the research on the health effects of informal care. Secondly, there are some issues of self-selection and endogeneity in the effect of informal care on the health of caregivers. Existing studies are more inclined to assess the correlation between the two, while the treatment of endogeneity is somewhat insufficient. In this paper, we will use propensity score matching (PSM) and instrumental variables (IV) estimation, respectively, on the basis of baseline regressions to minimize the impact of self-selection and endogeneity on the estimation results. Finally, existing research on the effect of spousal care on caregivers is more about the United States and European countries, with relatively limited research on China. This paper plans to explore the effect of spousal care on the health of middle-aged and older adult caregivers using internationally comparable CHARLS data, and to provide a supplement on spousal care perspectives based on Chinese situations.

### 2.2 Research hypothesis

In spousal care, the provision of care is physically and emotionally demanding for caregivers who are also middle-aged and older adult people (7). The long periods of intense caregiving put the physical and mental health of the caregivers under great stress (23). Long-term follow-up studies have shown no significant decline in cognitive function or self-reported health among US spousal caregivers (26). Studies using 2011–2015 CHARLS data have shown that individuals involved in spousal caregiving have more severe depressive symptoms than those not involved in spousal caregiving over the 4-year study period (27).

Based on the above analyses, two research hypotheses for this paper are proposed:

*H1*: Overall, the provision of spousal care does not have a significant effect on the physical health of middle-aged and older adult caregivers.

*H2*: Overall, the provision of spousal care has a negative effect on the mental health of middle-aged and older adult caregivers and the negative effect is stronger as the intensity of care increases.

Generally speaking, there is a strong gender division of care work, with women taking on more care work relative to men (10, 17, 28, 29). In addition, middle-aged and older adult people in rural areas are likely to be in relatively poorer health and have a higher proportion of needing to be cared for than their counterparts in urban areas (17), and the effect for caregivers will be greater. Therefore, this paper further proposes the third research hypothesis:

*H3*: The negative effect of the provision of spousal care on the health of caregivers varies across different groups; the negative effect is stronger for female and rural middle-aged and older adult caregivers.

What are the channels through which the effects of care work on health occur? Existing research suggests that increased social participation can alleviate depressive symptoms associated with intense care provision (27). On this basis, this paper considers that on the one hand, care provision negatively affects the health of caregivers by depleting their physical strength and reducing their disposable time, which further reduces their participation in physical exercise. On the other hand, the provision of spousal care will result in more consumption needs in terms of purchases of care materials, etc., thus increasing the total expenditure of the family, which in turn will have a negative effect on the health of the individuals by increasing their mental stress. Based on the above analysis, the fourth research hypothesis of this paper is proposed:

*H4*: The provision of spousal care can negatively affect the health of caregivers by reducing their frequency of exercise and increasing total household expenditure.

## 3 Data and methods

### 3.1 Data

The data in this paper come from the official combined Harmonized CHARLS database of the China Health and Retirement Longitudinal Study (CHARLS) 2011, 2013, 2015, and 2018. China Health and Retirement Longitudinal Study (CHARLS) is a large-scale interdisciplinary survey project jointly carried out by Wuhan University and Peking University. The survey covers 450 villages in 150 counties in 28 provinces (autonomous regions and municipalities directly under the central government). The interviewed individuals are 17,708 in CHARLS 2011, 18,612 in CHARLS 2013, 21,097 in CHARLS 2015 and 19,816 in CHARLS 2018, respectively. The database collects a set of high-quality microdata representing households and individuals of middle-aged and older adult Chinese people aged 45 years and older, and is one of the most detailed and richly sampled databases used to analyze the influence of spousal care on the health of middle-aged and older adult caregivers in this paper.

Harmonized CHARLS is integrated by Center for Economic and Social Research, University of Southern California based on CHARLS 2011, 2013, 2015, and 2018. This paper uses version D published in June, 2021 in the following descriptive and empirical analysis. In Harmonized CHARLS, if the variable relates to personal information, it has information on both the respondent as well as his/her spouse. Before descriptive and empirical analysis, this paper further deleted the following samples including the spouse of the individual “healthy and young” “do not know” “no help received” “missing” “unmarried” “separation” and “no response.” The numbers of deletions in each year were 3,171 in CHARLS 2011, 4,564 in CHARLS 2013, 5,299 in CHARLS 2015 and 5,048 in CHARLS 2018.

### 3.2 Methods

This paper explores whether there is a significant effect of spousal care on the health of middle-aged and older adult caregivers using a panel data fixed effects model. The model is constructed as follows:


(1) $$Health_{it}=\alpha +\beta *Spouse\_care_{it}+\gamma Z_{it}+\lambda_{i}+\theta_{t}+\mu_{it}$$

The explained variable $Health_{it}$ in Equation 1 is the health status of middle-aged and older adult caregivers, including physical health and mental health. Referring to relevant research (7, 11, 26, 30), physical health ${\mathrm{Shlta}}_{it}\;$ is measured by self-reported health of respondents. The question in CHARLS questionnaire is “Would you say your health is very good, good, fair, poor or very poor?” The answers are 1. Very good, 2. Good, 3. Fair, 4. Poor and 5. Very poor, respectively. The larger the value, the worse the physical condition.

For mental health, referring to the relevant research (7, 27, 30), mental health was measured by CESD10 score. After adjustment and summation of basic variables, the value of CESD10 score is between 0 and 30. If the value is greater than 10, it indicates that the individual has a tendency to depression. The questions in CHARLS questionnaire and calculations are as follows. The score is calculated by 10 questions. The contents are: “1.I was bothered by things that do not usually bother me.” “2. I had trouble keeping my mind on what I was doing.” “3.I felt depressed.” “4. I felt everything I did was an effort.” “5. I felt hopeful about the future.” “6. I felt fearful.” “7. My sleep was restless.” “8.I was happy.” “9. I felt lonely.” “10. I could not get ‘going’,”. Each of these questions have four options. There are “1. Rarely or none of the time” “2. Some or a little of the time” “3. Occasionally or a moderate amount of the time” and “4. Most or all of the time.” First, we reversed the scores of questions 5 and 8. Then, subtract 1 from the score of each option, that is, adjust the value from 1 to 4 to 0 to 3. Finally, the scores of 10 questions are summed up to get the CESD10 score to measure the mental status of middle-aged and older adult caregivers. Hence, the larger the value means that the mental status of the middle-aged and older adult people is worse. In the research samples of this paper, the proportion of middle-aged and older adult people whose value of $\mathrm{CESD}10_{it}$ less than or equal to 10 is 69.82%.

The key explanatory variable in Equation 1 is $Spouse\_care_{it}$ . Referring to the definition of spousal care in existing studies (7, 31) and combining the variable settings in Harmonized CHARLS database, this paper defines a spousal caregiver through the following two steps. First, the individual’s spouse needs help in at least one of the Activities of Daily Living (ADLs) or Instrumental Activities of Daily Living (IADLs). Activities of Daily Living (ADLs) include getting dressed, bathing, eating, getting into or out of bed, and using the toilet. Instrumental Activities of Daily Living (IADLs) include cooking, shopping for groceries, using the telephone, taking medication, and managing money. Second, when the individual’s spouse was asked, “Who most often helps you with these ADL and IADL difficulties?” The option “Spouse” was selected. If both conditions are met, the individual is recognized as a spousal caregiver. If the individual’s spouse does not need help, or if the spouse does not provide help when help is needed, the individual is identified as a non-spousal caregiver. The paper further takes into account the fact that there may be differences in the effect of different intensities of care on caregivers. Therefore, based on data availability, the intensity of care is further measured by the number of caregiving hours per month. $Z_{it}$ refer to individual- or household-level control variables, including age, gender, education, hukou, work status, health insurance, health of spouse, total household income, number of children, and financial support from children/grandchildren of the caregiver. $\lambda_{i}$ refers to individual fixed effects, $\theta_{t}$ refers to time-fixed effects, and $\mu_{it}$ is a random error term.

### 3.3 Descriptive analysis

Table 1 shows that the mean level of physical health of the study sample is 3.008, which is a general level of health, and the mean level of CESD10 scores is 8.158, which means no tendency to depression. The proportion of middle-aged and older adult people providing spousal care in the study sample is 17.2 percent. The age of the study sample in this paper ranges from 45 to 86 years old with a mean age of 60.38 years old. Gender is a 0 or 1 binary dummy variable, with 0 indicating male and 1 indicating female. The education level is divided into four categories: below primary school, primary school, junior high school and senior high school and above. The proportion of samples with the education level of below primary school is 42.9%. The proportion with primary school is 24.3%. The proportion with junior high school is 21.6%. And the proportion with senior high school and above is 11.2%, presenting the higher the education level, the lower the proportion of the study samples. Hukou is a 0 or1 binary dummy variable, where 0 represents agricultural hukou and 1 represents non-agricultural hukou. Individual work status is a binary dummy variable of 0 or 1, with 0 indicating that the surveyed individual is “unemployed, retired or never worked” and 1 indicating that the surveyed individual is “engaging in agricultural work, non-agricultural employed work, non-agricultural self-employed work, or non-agricultural unpaid family business work.” Health insurance is a 0 or 1 binary dummy variable, with 0 indicating that the individual is not covered by public health insurance and 1 indicating that the individual is covered by public health insurance. Health of spouse is a sequential variable taking values from 1 to 5, with the larger the value, the worse the health of the spouse. Total household income is a continuous variable with a mean value of 31,059 yuan. Number of children is an ordinal variable taking values between 0 and 10, with a mean number of children of 2.69. Financial support is a variable measuring wealth transfer from children/grandchildren, with a mean value of 3,220 yuan in the study sample.

**Table 1:** Descriptive statistics of the main variables.

Variable type and variable name	N	Mean	S.D.	Min	Max
Explained variable					
Shlta	24,873	3.008	0.954	1	5
CESD10	24,358	8.158	6.170	0	30
Key explanatory variable					
Spousal care	24,873	0.172	0.378	0	1
Individual characteristics of caregivers					
Age	24,873	60.38	8.634	45	86
Gender	24,873	0.501	0.500	0	1
Education below primary school	24,873	0.429	0.495	0	1
Education of primary school	24,873	0.243	0.429	0	1
Education of junior high school	24,873	0.216	0.411	0	1
Education of senior high school and above	24,873	0.112	0.315	0	1
Hukou	24,873	0.207	0.406	0	1
Work status	24,873	0.634	0.482	0	1
Health insurance	24,873	0.951	0.215	0	1
Household characteristics of caregivers					
Health of spouse	24,873	2.995	0.959	1	5
Total household income (RMB)	24,873	31,059	36,665	130	227,800
Number of children	24,873	2.692	1.330	0	10
Financial support (RMB)	24,873	3,220	5,544	0	40,000

Age and Financial support are post-tailored by 1 percent. Total household income has been rounded off by 1 percent.

## 4 Analysis of empirical results

### 4.1 Baseline regression analysis

Table 2 presents the results of baseline regressions. Column (1) and column (4) present the results of the regression with only the key explanatory variable added. It can be seen that at the 1% significance level, spousal care significantly increases caregivers’ self-reported health scores and CESD10 scores, with significant negative effects on both physical and mental health. Further, under the control of the caregiver’s age, gender, education, hukou, work status, health insurance, health of spouse, total household income, number of children, financial support from children/grandchildren, it can be seen from column (2) and column (5), spousal care has a significant positive effect on the physical health of middle-aged and older adult caregivers, but has a significant negative effect on their mental health. For physical health, the results are unstable. Therefore, this paper further controls all control variables, individual fixed effects and time fixed effects, and the results of column (3) and column (6) show that spousal care has no significant effects on the physical health of middle-aged and older adult caregivers, but has a significant negative effects on their mental health. Hypotheses 1 and 2 of this paper are verified. The CESD10 scores of middle-aged and older adult caregivers increase by 0.322 relative to those who do not provide spousal care, which is 3.95% of the total sample mean.

**Table 2:** Baseline regression results of the effects of spousal care on the health of middle-aged and older adult caregivers.

	(1)	(2)	(3)	(4)	(5)	(6)
Variables	Shlta	Shlta	Shlta	CESD10	CESD10	CESD10
Spousal care	0.096^***^	−0.034^**^	−0.001	1.405^***^	0.909^***^	0.322^**^
	(0.016)	(0.016)	(0.022)	(0.105)	(0.105)	(0.138)
Control variables	No	Yes	Yes	No	Yes	Yes
Individual FE	No	No	Yes	No	No	Yes
Time FE	No	No	Yes	No	No	Yes
Observations	24,873	24,873	24,873	24,358	24,358	24,358
R^2^	0.000	0.011	0.014	0.001	0.004	0.013
Number of ID	14,271	14,271	14,271	14,005	14,005	14,005

∗∗∗, ∗∗, and ∗ represent significance at the 1, 5, and 10 percent levels, respectively. Heteroskedasticity robust standard errors are in parentheses.

### 4.2 Robustness tests

First, this paper restricts the study samples to older caregivers aged 60 and above. The regressions in columns (1) and (3) of Table 3 show that spousal care does not have a significant effect on the physical health of older caregivers aged 60 and above; however, it significantly reduces their mental health level, consistent with the results of the baseline regressions. In addition, this paper changes the way of measuring physical health and mental health in the baseline regressions. The new variable $\mathrm{poor}\_{\mathrm{SRH}}_{it}$ is defined as a 0 or1 binary dummy variable to measure the physical condition. When ${\mathrm{shlta}}_{it}$ takes the value of 1, 2 or 3, $\mathrm{poor}\_{\mathrm{SRH}}_{it}$ takes the value of 0, that is, the individual’s physical condition is good. When ${\mathrm{shlta}}_{it}$ takes the value of 4 or 5, $\mathrm{poor}\_{\mathrm{SRH}}_{it}$ takes the value of 1, that is, the individual’s physical condition is poor. Define the new variable ${\mathrm{depression}}_{it}$ as a 0 or 1 binary dummy variable to measure mental condition. When ${\mathrm{CESD}}_{it}$ is less than or equal to 10, ${\mathrm{depression}}_{it}$ takes the value of 0, that is, the individual does not have depressive tendency. When ${\mathrm{CESD}}_{it}$ is greater than 10, ${\mathrm{depression}}_{it}$ takes the value of 1, that is, the individual has depressive tendency. The regressions in columns (2) and (4) show that spousal care still does not have a significant effect on the new definition of physical condition, but increases the likelihood of an individual’s new definition of poor mental condition at the 10 percent significance level. The conclusions remain consistent with the baseline regressions.

**Table 3:** Robustness test of the effects of spousal care on the health of middle-aged and older adult caregivers.

	(1)	(2)	(3)	(4)
Variables	Shlta	Poor_SRH	CESD10	Depression
Spousal care	0.006	−0.001	0.417^**^	0.021^*^
	(0.029)	(0.011)	(0.179)	(0.012)
Control variables	Yes	Yes	Yes	Yes
Individual FE	Yes	Yes	Yes	Yes
Time FE	Yes	Yes	Yes	Yes
Observations	13,099	24,873	12,823	24,358
R^2^	0.016	0.012	0.014	0.010
Number of ID	7,926	14,271	7,760	14,005

∗∗∗, ∗∗, and ∗ represent significance at the 1, 5, and 10 percent levels, respectively. Heteroskedasticity robust standard errors are in parentheses.

### 4.3 Endogenous treatment

#### 4.3.1 Sample selection correction

In the process of studying the effects of spousal care on the health, spousal caregivers themselves may be middle-aged and older adult people in better health, so there is some sample self-selection problem. This paper further analyses the relationship using propensity score matching (PSM). First, a balance test between the two groups of middle-aged and older adult samples that provide and do not provide spousal care is conducted. The results show that the proportions of bias in control variables of the samples in both groups are reduced to less than 5% after matching. Meanwhile, the absolute value of the *t*-value of the matched samples is also significantly reduced, and the *p*-values of the significance tests are greater than 0.05; indicating that the matched samples pass the PSM balance test at the 5% significance level. The results of the balance test can be found in the Supplementary materials section at the end of this paper.

In this paper, three matching methods, kernel matching, radius matching and k-nearest neighbor matching, are used to explore the effects of spousal care on the health of middle-aged and older adult caregivers. In this case, k-nearest neighbor matching is performed for one-to-one and one-to-two matching, respectively. Table 4 shows the estimation results with physical health as the explained variable. The pre-matching gap between the treatment and control groups is 0.143, indicating that the middle-aged and older adult people who provided spousal care are in worse physical condition than those who do not and the results are significant. After matching the gap narrows to −0.031 to −0.022 and none of the results are significant anymore. Thus, after taking the self-selection problem into account, there is no evidence of a significant effect of spousal care on the physical health of middle-aged and older adult caregivers, consistent with the results of the baseline regressions.

**Table 4:** Estimation results of the effects of spousal care on caregivers’ physical health by PSM method.

	Care	Non-care	Difference	Standard error	*T*-values
Before matching	3.127	2.984	0.143	0.016	8.96***
Kernel matching	3.127	3.149	−0.022	0.018	−1.24
Radius matching	3.126	3.151	−0.025	0.018	−1.40
One-to-one nearest neighbor matching	3.127	3.158	−0.031	0.024	−1.31
One-to-two nearest neighbor matching	3.127	3.156	−0.029	0.021	−1.38

Kernel matching uses a default bandwidth of 0.06, and the value of ATT varies very little when other parameters are used for matching. In radius matching, the range of the radius is generally no more than 0.25 times of the standard deviation of the samples of propensity scores, and based on prudent considerations, the range of the radius is further narrowed down to be set at 0.03. ***, **, and * represent significance at the 1, 5, and 10 percent levels, respectively.

Table 5 shows the estimation results with mental health as the explained variable. Before matching, the difference between the treatment and control groups is 1.977, indicating that the mental health of middle-aged and older adult caregivers is worse, and the result is significant. After matching, the gap narrows to 1.074–1.248, and the results remain significant. This suggests that middle-aged and older adult caregivers do experience negative shocks to their mental health while providing spousal care. The PSM estimation results show that, on average, the CESD10 scores of middle-aged and older adult people who provide spousal care are higher by 1.173 relative to those who do not provide spousal care, which is 14.37 percent of the full sample mean.

**Table 5:** Estimation results of the effects of spousal care on caregivers’ mental health by PSM method.

	Care	Non-care	Difference	Standard error	*T*-values
Before matching	9.791	7.814	1.977	0.103	19.11***
Kernel matching	9.793	8.546	1.248	0.122	10.19***
Radius matching	9.797	8.555	1.242	0.123	10.10***
One-to-one nearest neighbor matching	9.791	8.716	1.074	0.165	6.52***
One-to-two nearest neighbor matching	9.791	8.665	1.126	0.146	7.73***

Kernel matching uses a default bandwidth of 0.06, and the value of ATT varies very little when other parameters are used for matching. In radius matching, the range of the radius is generally no more than 0.25 times of the standard deviation of the samples of propensity scores, and based on prudent considerations, the range of the radius is further narrowed down to be set at 0.03. ***, **, and * represent significance at the 1, 5, and 10 percent levels, respectively.

#### 4.3.2 The problem of omitted variables

In addition to the sample self-selection problem, there may be unobservable factors that affect both the choice of spousal care and the health of the middle-aged and older adult people. Therefore, appropriate instrumental variables for spousal care need to be selected. With reference to the instrumental variables in existing research (31), this paper selects spousal self-care ability as an instrumental variable to explore the effect of spousal care on the health of middle-aged and older adult caregivers. On the one hand, there is a positive correlation between a spouse’s ability to take care of himself/herself and whether or not to be cared for; the worse the ability to take care of himself/herself, the higher the likelihood that spousal care will be provided. On the other hand, after controlling for the health of the spouse, the effect of spousal self-care ability on the health of the caregiver is relatively small and basically satisfies the exogeneity assumption of the instrumental variable. In the Harmonized CHARLS database, self-care ability includes self-care ability of ADLs and self-care ability of IADLs. Self-care ability of ADLs is measured by the ability to care for oneself in five activities of daily living: dressing, bathing, eating, getting up and out of bed and using the toilet. If an individual’s spouse has difficulty with one of the above activities, a value of 1 is assigned, otherwise a value of 0 is assigned. The number of difficulties encountered by an individual’s spouse is summed up to obtain the individual’s self-care ability of ADLs, which is a variable with a value between 0 and 5; the higher the value, the poorer the individual’s self-care ability of ADLs. Self-care ability of IADLs is measured by the ability to care for oneself in five instrumental activities of daily living: cooking, shopping for groceries, using the telephone, taking medication and managing money; which is also a variable with a value between 0 and 5 and are defined in the same way as self-care ability of ADLs.

In this paper, we run 2SLS regressions using spousal self-care ability of ADLs together with self-care ability of IADLs as instrumental variables for spousal care. Columns (1) and (2) of Table 6 show the results of the first-stage regressions with physical health and mental health as explained variables, respectively. It can be seen that there is a positive and significant effect of both self-care ability of ADLs and self-care ability of IADLs on the choice of spousal care under the control of all control variables, individual fixed effects and time fixed effects; meanwhile the *F*-values for the one-stage are 438.07 and 438.09 respectively, both significantly greater than 10, ruling out the possibility of weak instrumental variables. Columns (3) and (4) show the results of the second-stage regressions, respectively, and it can be seen that spousal care still does not have a significant effect on the physical health of middle-aged and older adult caregivers after endogeneity is taken into account; however, it does significantly increase their CESD10 scores, and negatively affects their mental health; consistent with the findings of the baseline regressions.

**Table 6:** Estimation results of the effects of spousal care on the health of caregivers by instrumental variables estimation.

	First-stage regression		Second-stage regression	
	(1)	(2)	(3)	(4)
Variables	Spousal care	Spousal care	Shlta	CESD10
Self-care ability of ADLs	0.036***	0.032***		
	(0.011)	(0.011)		
Self-care ability of IADLs	0.253***	0.255***		
	(0.010)	(0.010)		
Spousal care			0.058	0.895***
			(0.054)	(0.344)
Control variables	Yes	Yes	Yes	Yes
Individual FE	Yes	Yes	Yes	Yes
Time FE	Yes	Yes	Yes	Yes
Observations	9,806	9,726	9,806	9,726
F-values	438.07	438.09	6.21	7.95
Number of ID	4,384	4,348	4,384	4,348

∗∗∗, ∗∗, and ∗ represent significance at the 1, 5, and 10 percent levels, respectively. Heteroskedasticity robust standard errors are in parentheses.

### 4.4 Heterogeneity analysis

The results of the sub-sample regressions in Table 7 show that the provision of spousal care does not have a significant effect on the physical health of both male and female, urban and rural middle-aged and older adult caregivers. This further supports the findings in the baseline regressions that the provision of spousal care does not have a significant effect on the physical health of middle-aged and older adult caregivers.

**Table 7:** Heterogeneity analysis of spousal care on the physical health of middle-aged and older adult caregivers.

	(1)	(2)	(3)	(4)	(5)
Variables	All	Male	Female	Rural	Urban
Spousal care	−0.001	−0.014	0.010	−0.008	0.060
	(0.022)	(0.029)	(0.034)	(0.025)	(0.052)
Control variables	Yes	Yes	Yes	Yes	Yes
Individual FE	Yes	Yes	Yes	Yes	Yes
Time FE	Yes	Yes	Yes	Yes	Yes
Observations	24,873	12,418	12,455	19,712	5,161
R^2^	0.014	0.019	0.014	0.014	0.018
Number of ID	14,271	7,140	7,131	11,275	3,169

∗∗∗, ∗∗, and ∗ represent significance at the 1, 5, and 10 percent levels, respectively. Heteroskedasticity robust standard errors are in parentheses.

The sub-sample regression results in Table 8 show that the provision of spousal care has a greater negative effect on the mental health of female middle-aged and older adult caregivers than the overall average, while there is no significant effect on male middle-aged and older adult caregivers. Generally speaking, the traditional gender division of labor and role consciousness make women take on more unpaid care and domestic work, while men are more involved in paid market work, and the proportion and duration of their involvement in caregiving are relatively smaller. At the same time, the traditional rules of marital matching take on the characteristic of “men are older and women are younger,” and men will enter the stage of advanced age and disability at an earlier stage than younger spouses; therefore, the probability of women caring for their husbands may be higher and the duration of caregiving may be longer; presenting the conclusion that caregiving has a greater negative effect on the mental health of female caregivers.

**Table 8:** Heterogeneity analysis of spousal care on the mental health of middle-aged and older adult caregivers.

	(1)	(2)	(3)	(4)	(5)
Variables	All	Male	Female	Rural	Urban
Spousal care	0.322^**^	0.153	0.546^**^	0.401^***^	−0.311
	(0.138)	(0.171)	(0.229)	(0.154)	(0.305)
Control variables	Yes	Yes	Yes	Yes	Yes
Individual FE	Yes	Yes	Yes	Yes	Yes
Time FE	Yes	Yes	Yes	Yes	Yes
Observations	24,358	12,155	12,203	19,316	5,042
R^2^	0.013	0.011	0.017	0.013	0.019
Number of ID	14,005	6,995	7,010	11,074	3,096

∗∗∗, ∗∗, and ∗∗represent significance at the 1, 5, and 10 percent levels, respectively. Heteroskedasticity robust standard errors are in parentheses.

In the regressions of rural and urban samples, the provision of spousal care has a greater negative effect on the mental health of rural middle-aged and older adult caregivers than the overall average but does not have a significant effect on the mental health of urban caregivers. On the one hand, this may be due to the fact that middle-aged and older adult people in urban areas have better health care systems and more leisure and recreational options to alleviate negative emotions, reducing the negative effects of caregiving on the mental health. On the other hand, it may be due to the fact that the samples of the study in this paper are more rural middle-aged and older adult people, and the samples of urban middle-aged and older adult people are relatively small. Therefore, it is not found for the time being that the provision of spousal care has a significant negative effect on the mental health of urban middle-aged and older adult caregivers. The above findings validate Hypothesis 3 of this paper.

### 4.5 Mechanism analysis

Through what channels does the effect of spousal care on the health of middle-aged and older adult caregivers actually occur? This paper attempts to explain this in the mechanism analysis section. One of the mechanism variables selected for this paper is the number of days of weekly exercise for caregivers. As can be seen from the regression in column (1) of Table 9, the provision of spousal care reduces the number of days of weekly exercise for middle-aged and older adult caregivers at the 10 percent significance level, under the control of all the control variables, individual fixed effects and time fixed effects. Typically, exercise improves the mental health of middle-aged and older adult caregivers through channels such as increasing social interaction and relieving mental stress; whereas the provision of care reduces the frequency of exercise for caregivers, which in turn can have a negative effect on their mental health.

**Table 9:** Mechanism analysis of spousal care on the health of middle-aged and older adult caregivers.

	(1)	(2)
Variables	Days of weekly exercise	Household’s non-food expenditure
Spousal care	−0.236*	0.082**
	(0.129)	(0.037)
Control variables	Yes	Yes
Individual FE	Yes	Yes
Time FE	Yes	Yes
Observations	14,796	22,591
R^2^	0.017	0.034
Number of ID	10,699	13,436

∗∗∗, ∗∗, and ∗∗represent significance at the 1, 5, and 10 percent levels, respectively. Heteroskedasticity robust standard errors are in parentheses.

Another mechanism variable selected for this paper is the household’s non-food expenditure in the previous year. Column (2) of Table 9 shows that the provision of spousal care increases households’ non-food expenditures at the 5 percent significance level, under the control of all the control variables, individual fixed effects and time fixed effects. Households providing spousal care are likely to have more consumption needs, such as for the purchase of care materials, and thus higher non-food expenditures, than households not providing care. And with a certain total household income, financial support from children/grandchildren, the increase in expenditure will increase the mental stress of middle-aged and older adult caregivers, which in turn will have a negative effect on their mental health. Hypothesis 4 of this paper is verified.

### 4.6 Further analysis

In analyzing the effects of spousal care on the health of middle-aged and older adult caregivers, this paper further considers whether there are differences in the effects of different care intensities on the health of caregivers? With reference to existing research on the measurement of care intensity (32–34) and based on the availability of data, this paper further calculates the number of hours of care per month for individuals providing spousal care as a measurement of spousal care intensity. The regressions in columns (1) and (2) of Table 10 show that, under the control of all the control variables, individual fixed effects and time fixed effects, an increase in the number of care hours per month does not have a significant effect on the physical health of middle-aged and older adult caregivers. However, as the number of care hours per month increases, middle-aged and older adult caregivers’ CESD10 scores increase significantly, suggesting a stronger negative effect on their mental health. Hypothesis 2 of this paper is further tested.

**Table 10:** Estimation results of the effects of spousal care intensity and “being able to help the spouse with his/her future ADL needs” on the health of middle-aged and older adult caregivers.

	(1)	(2)	(3)	(4)
Variables	Shlta	CESD10	Shlta	CESD10
Spousal care intensity	0.049	0.516**		
	(0.032)	0.233		
Spousal care in the			−0.024	−0.317**
future			(0.024)	(0.154)
Control variables	Yes	Yes	Yes	Yes
Individual FE	Yes	Yes	Yes	Yes
Time FE	Yes	Yes	Yes	Yes
Observations	2,791	2,758	16,638	16,517
R^2^	0.027	0.059	0.013	0.018
Number of ID	2,408	2,378	11,584	11,505

∗∗∗, ∗∗, and ∗ represent significance at the 1, 5, and 10 percent levels, respectively. Heteroskedasticity robust standard errors are in parentheses.

On the basis of analyzing the effects of realistic spousal care on the health of the caregivers, this paper further considers whether there would be an effect on the health of a caregiver if the individual was able to provide help for his/her spouse’s future ADL needs? Based on the availability of data, this paper further regresses with the binary dummy variable of ‘being able to help the spouse with his/her future ADL needs’ as the key explanatory variable. The results in columns (3) and (4) of Table 10 show that “being able to help the spouse with his/her future ADL needs” does not have a significant effect on the physical health of middle-aged and older adult caregivers. However, it will significantly reduce their CESD10 scores and have a significant positive effect on their mental health. Generally speaking, when an individual realizes that he/she can help his/her spouse with the ADLs in the future, it means that his/her health and schedule are at his/her disposal, and the probability of uncertainty in future family care is reduced, thus reducing his/her mental stress and having a positive effect on the mental health.

## 5 Conclusions and recommendations

The research in this paper shows that the provision of spousal care in the middle-aged and older adult groups has had a negative effect on their own mental health, which in turn has challenged the needs of the healthcare system and the goals of “Healthy Aging.” And there is no evidence that spousal care has had an effect on the physical health of middle-aged and older adult caregivers. The conclusions above remain robust after narrowing the research samples to older caregivers aged 60 and above, changing the measurements of physical and mental health, and using instrumental variables (IV) estimation and propensity score matching (PSM) regressions on the research samples.

The results of the sub-sample regressions indicate that spousal care has a greater negative effect on the mental health of female, rural middle-aged and older adult caregivers than the overall average; while it does not have a significant effect on male, urban middle-aged and older adult caregivers. Spousal care has a negative effect on the mental health of middle-aged and older adult caregivers by reducing the number of days of weekly exercise and increasing the family’s non-food expenditure in the previous year. Further analyses of different care intensities show that the provision of spousal care has a greater negative effect on the mental health as the care intensity increases; and that being able to help the spouse with his/her future ADL needs significantly improves the mental health of middle-aged and older adult caregivers.

Despite these interesting results, our research has some limitations that must be kept in mind. On the one hand, in exploring the influence of spousal care on the health of caregivers, there is a certain endogeneity problem. This paper comprehensively uses the analysis methods of propensity score matching (PSM) and instrumental variables (IV) estimation to alleviate the influence of endogenous problems on the estimation results. However, in the existing research, the application of instrumental variables (IV) estimation is limited, and future research could consider whether more exogenous instrumental variables can be found to estimate the health effects of spousal care on caregivers. On the other hand, due to the limitation of data, the proportion of middle-aged and older adult people in rural areas in this paper is relatively high, so the research on urban middle-aged and older adult groups is relatively less representative. If there are data for urban middle-aged and older adult groups in the later period, follow-up research can strengthen the research in this area.

Although to some extent spousal care has improved the situation in China, where child care does not meet care needs, it has had a certain degree of negative effect on the mental health of spouses who are also middle-aged or older adult people. Therefore, the community service system for the older adult should be optimized, and more attention and support should be given to families in which both spouses need care. Secondly, colleges and training institutions should be encouraged to offer courses in older adult care and geriatric nursing, so as to expand the pool of specialists and meet the urgent and growing demand for health care for the older adult. Finally, share innovative models of older adult care. Use emerging technologies such as the Internet of Things, 5G and big data to strengthen the connection between medical resources and older adult care resources to achieve the goal of “Healthy Aging.”

## Data Availability

Publicly available datasets were analyzed in this study. This data can be found at: China Health and Retirement Longitudinal Study (CHARLS) (https://charls.pku.edu.cn).
