# Supplementary material for: The influence of spousal care on the health of middle-aged and older adult caregivers in China—empirical analysis based on CHARLS data
Source: Front Public Health. 2025 Feb 6;13:1496637. doi: 10.3389/fpubh.2025.1496637 (PMC11839437; doi:10.3389/fpubh.2025.1496637)
Supplement: Supplementary file 1 [file Supplementary_file_1.docx]

Appendix. The results of balance test before and after spousal care matching

| Variables | Samples | Mean | | Standard  deviation（%） | Deviation  Reduction （%） | T-test | |
| --- | --- | --- | --- | --- | --- | --- | --- |
|  |  | Care | Non- care |  |  | T-values | P-values |
| Age | Before | 63.522 | 59.691 | 45.4 | 99.7 | 26.74 | 0.000 |
|  | After | 63.519 | 63.53 | -0.1 |  | -0.06 | 0.951 |
| Gender | Before | 0.423 | 0.517 | -19.0 | 96.0 | -11.20 | 0.000 |
|  | After | 0.423 | 0.419 | 0.8 |  | 0.35 | 0.725 |
| Primary school | Before | 0.242 | 0.245 | -0.6 | 76.3 | -0.37 | 0.711 |
|  | After | 0.242 | 0.243 | -0.1 |  | -0.07 | 0.945 |
| Junior high | Before | 0.184 | 0.181 | -9.7 | 92.6 | -5.61 | 0.000 |
| school | After | 0.184 | 0.180 | 0.7 |  | 0.34 | 0.730 |
| Senior high  school and above | Before | 0.086 | 0.118 | -10.3 | 99.6 | -5.84 | 0.000 |
|  | After | 0.086 | 0.087 | -0.0 |  | -0.02 | 0.983 |
| Hukou | Before | 0.168 | 0.215 | -12.1 | 94.9 | -6.96 | 0.000 |
|  | After | 0.168 | 0.165 | 0.6 |  | 0.30 | 0.762 |
| Total household  income | Before | 8.992 | 9.568 | -37.6 | 97.3 | -22.02 | 0.000 |
|  | After | 8.993 | 8.977 | 1.0 |  | 0.47 | 0.640 |
| Work status | Before | 0.629 | 0.640 | -2.4 | 92.2 | -1.42 | 0.155 |
|  | After | 0.629 | 0.628 | 0.2 |  | 0.09 | 0.932 |
| Financial support | Before | 5.828 | 4.9 | 24.8 | 97.4 | 14.24 | 0.000 |
|  | After | 5.827 | 5.803 | 0.6 |  | 0.31 | 0.754 |
| Health insurance | Before | 0.955 | 0.951 | 2.2 | 65.8 | 1.26 | 0.209 |
|  | After | 0.955 | 0.954 | 0.7 |  | 0.35 | 0.730 |
| Health of spouse | Before | 3.585 | 2.869 | 77.5 | 94.4 | 46.06 | 0.000 |
|  | After | 3.585 | 3.545 | 4.4 |  | 2.07 | 0.039 |
| Number of children | Before | 3.04 | 2.615 | 31.1 | 96.0 | 19.14 | 0.000 |
|  | After | 3.039 | 3.056 | -1.3 |  | -0.54 | 0.586 |
| The year 2013 | Before | 0.288 | 0.222 | 15.2 | 97.0 | 9.26 | 0.000 |
|  | After | 0.288 | 0.290 | -0.5 |  | -0.20 | 0.840 |
| The year 2015 | Before | 0.191 | 0.157 | 9.1 | 95.5 | 5.50 | 0.000 |
|  | After | 0.191 | 0.189 | 0.4 |  | 0.18 | 0.857 |
| The year 2018 | Before | 0.298 | 0.313 | -3.4 | 59.2 | -1.98 | 0.047 |
|  | After | 0.298 | 0.291 | 1.4 |  | 0.64 | 0.523 |

Note: Total household income and financial support are the values after logarithmic processing.

|  |
| --- |
|  |
